# Promoting patient-centred care in the management of allergic rhinitis in Asia-Pacific countries^☆^

**DOI:** 10.1016/j.waojou.2024.100952

**Published:** 2024-08-22

**Authors:** Hiroshi Chantaphakul, De Yun Wang, Tran Thi Thuy Hang, Khizuan Abdul Kadir, Hoang Thi Lam, Cecilia Gretchen Navarro-Locsin, Sira Nanthapisal, Danilo Poblete, Pongsakorn Tantilipikorn, Wong Hui Tong, Dinesh Nagrale, Michaela Lucas

**Affiliations:** a Division of Allergy and Clinical Immunology, Department of Medicine, Faculty of Medicine, Chulalongkorn University, Bangkok, Thailand; b Department of Otolaryngology, Yong Loo Lin School of Medicine, National University of Singapore, Singapore; c ORL Department, Tam Anh General Hospital, Ho Chi Minh City, Viet Nam; d Hospital Pakar An Nur, Bandar Baru Bangi, Malaysia; e Unit of Allergy and Clinical Immunology, University Medical Center, Ho Chi Minh City, Viet Nam; f Philippine Children's Medical Center, Philippines, and St. Luke's Medical Center, Quezon City, Philippines; g Division of Allergy, Immunology, and Rheumatology, Thammasat University, Pathumthani, Thailand; h AMSI Doctors Medical Center, Calamba, Philippines; i Center of Research Excellence in Allergy & Immunology, Faculty of Medicine, Siriraj Hospital, Thailand; j Sunway Medical Centre, Kuala Lumpur, Malaysia; k Menarini Asia-Pacific Pte Ltd, Singapore; l Department of Immunology, QE Medical Centre, Sir Charles Gairdner Hospital, Perth Children's Hospital, University of Western Australia, Perth, Australia; m Medical School, University of Western Australia, Perth, Australia

**Keywords:** Rhinitis, Allergic, Allergy and immunology, Asia, Patient-centred care, Decision-making, Shared

## Abstract

**Background:**

Allergic rhinitis (AR) has a high burden of disease in the Asia-Pacific region (APAC). Although guidelines provide recommendations regarding the diagnosis and treatment of AR, it is increasingly being recognised that there are gaps in their implementation. Patient-centred care involves accounting for the specific needs and desires of patients as well as including the patient in the decision-making process, and this may provide a means to reduce these gaps and consequently the burden of AR.

**Methods:**

A group of 11 experts in immunology and otorhinolaryngology from APAC provided information regarding their practices and experiences in the management of AR through an online survey. The group then discussed the barriers and solutions for the implementation of patient-centred care across the patient journey in a face-to-face meeting.

**Results:**

Key barriers to the implementation of patient-centred care for AR in APAC included a lack of patient awareness of the condition and treatment options, low adherence to treatments, financial constraints for patients, and time constraints for physicians. The solutions proposed include improving the knowledge of the patients about their conditions, the use of shared decision-making, the consideration of patient characteristics when choosing treatments, and the use of outcome measures to aid the optimisation of patient care. We provide specific recommendations for clinical practice.

**Conclusion:**

A greater focus on patient-centred approaches has the potential to improve the management of AR in APAC. More emphasis should be placed on each patient's specific health needs and desired outcomes.

## Introduction

Patients with allergic rhinitis (AR) typically experience sneezing, rhinorrhoea, nasal obstruction, and itching due to an immunoglobulin E (IgE)-mediated hypersensitivity response.^1^ AR is also commonly associated with ocular symptoms such as itchy, red, or watering eyes in the form of allergic rhinoconjunctivitis.^2^ Allergic rhinitis can be classified into intermittent AR, with symptoms lasting less than 4 days per week or less than 4 consecutive weeks, or persistent AR, with symptoms lasting more than 4 days per week for at least 1 month.^1^ Traditionally, AR has also been classified as seasonal or perennial, depending on patterns of allergen exposure.^1^ A survey of 33,378 households in the Asia-Pacific region (APAC), including Australia, China, Hong Kong, Malaysia, Singapore, Taiwan, Vietnam, and the Philippines found that 8.7% of respondents have physician-diagnosed AR, with 63% of these reporting that they have seasonal AR.^3^ However, prevalence values for individual countries vary widely, ranging from 2.5% in the Philippines,^3^ to 42.6–57.4% in Thailand^4^^,^^5^ (Table 1). The proportion of patients reporting symptoms that occur seasonally, as opposed to throughout the year, also differs between countries, ranging from 24% of patients with AR in Singapore to 85% of those in China.^3^

**Table 1:** Reported prevalences of allergic rhinitis for Asia-Pacific countries.

Country	Prevalence	Type of study	Source
APAC^a^	8.7%	Telephone and in-person screening of 33,378 households.	Katelaris et al., 2011^3^
Australia	13.2%	Telephone screening of 3534 households	Katelaris et al., 2011^3^
Australia	46% (without confirmation by skin tests) 31.8% (with confirmation by skin tests)	Single-centre survey in Melbourne using the ECRHS screening questionnaire (549 respondents)	Bousquet et al., 2008^37^
China	9.8% (paediatric)	Cross-sectional survey in eight metropolitan cities using the ISAAC questionnaire (23,791 respondents)	Li et al., 2011^38^
China	34.3%	Survey using a standardized questionnaire in four major cities in western China (29,985 respondents)	Shen et al., 2011^39^
China	16.4%	Cases with signs/symptoms of AR in face-to-face surveys of 5010 individuals	Wang et al., 2011^40^
China	9.1%	Telephone screening of 19,580 households	Katelaris et al., 2011^3^
Hong Kong	4.2%	Telephone screening of 2118 households	Katelaris et al., 2011^3^
South Korea	18.9% (6–7 years of age) 19.2% (13–14 years)	Cross sectional survey of 4003 children aged 6–7 years and 4112 children aged 13–14 years	Ahn et al., 2011^41^
Malaysia	7.1%	In-person screening of 491 households	Katelaris et al., 2011^3^
Philippines	2.5%	In-person screening of 1285 households	Katelaris et al., 2011^3^
Singapore	4.9%	In person screening of 2002 households	Katelaris et al., 2011^3^
Taiwan	9.6%	Telephone screening of 1780 households	Katelaris et al., 2011^3^
Thailand	42.6%	Cumulative prevalence of rhinitis symptoms in a survey of school children in Khon Kaen, Northeastern Thailand (2119 6–7 years of age; 2956 13–14 years of age), using a Thai version of the ISAAC questionnaire	Teeratakulpisarn et al., 2004^4^
Thailand	57.4%	Survey of 2693 university students using the ISAAC questionnaire	Uthaisangsook et al., 2007^5^
Vietnam	29.6% (Hoankiem) 10% (Bavi)	Cross sectional survey, using the FinEsS questionnaire, in Hoankiem (2115 respondents) and Bavi (3667 respondents)	Lam et al., 2011^42^
Vietnam	12.3%	In-person screening of 2588 households	Katelaris et al., 2011^3^

Abbreviations: APAC, Asia-Pacific; ECRHS, European Community Respiratory Health Survey; ISAAC, International Study of Asthma and Allergies in Childhood.

a APAC includes data from Australia, China, Hong Kong, Malaysia, the Philippines, Singapore, Taiwan, and Vietnam

AR is associated with a substantial burden to the patient.^3^^,^^6^, ^7^, ^8^ Amongst patients in APAC, 38% experience a moderate-to-severe impact on their daily life.^3^ A study in Thailand found that quality of life (QoL) scores in all domains of the Rand 36-Item Short Form Survey Instrument (SF-36) questionnaire decrease as the severity of AR increases, with bodily pain, general health, and vitality the most severely affected.^7^ The symptoms of AR also have an impact on sleep, with >70% of those in APAC with self-reported AR reporting being at least somewhat troubled by sleep issues.^3^ Additionally, 46% of patients in APAC report that AR prevents them from performing well at work or school, with a >20% reduction in productivity when their symptoms are at their most severe.^3^ Similarly, in a global survey, AR sufferers reported missing an average of approximately half a day of work in the last 7 days due to AR.^8^

It is being increasingly recognised that there are gaps between published guidelines and their implementation in AR management.^9^ Real-world evidence has also shown that patient behaviour differs from that assumed in guidelines, with patients often self-medicating, not being consistently adherent, and tending to take medications on an ad hoc basis, only when symptoms are severe.^10^ One potential solution to improve AR management and reduce some of its associated burden is to use patient-centred approaches when implementing the recommendations of international or local guidelines. Patient-centred care involves accounting for the patient's specific health needs and desired health outcomes when making healthcare decisions.^11^ Shared decision making (SDM) is a key component of this approach, in which physicians work with patients to make informed decisions regarding diagnostic tests and treatment approaches.^12^ Previous studies have demonstrated that patient-centred approaches have numerous benefits, including improved outcomes and patient satisfaction,^13^ and reductions in referrals and diagnostic tests.^14^ The use of SDM has also been shown to improve adherence and clinical outcomes compared with either usual care or clinician decision-making in other disease areas (eg, asthma).^15^ In recent years, SDM has received attention in AR care, particularly in the context of decisions regarding allergen immunotherapy (AIT) where choices between sublingual and subcutaneous therapy may depend on patient preferences and long-term adherence is important.^12^^,^^16^, ^17^, ^18^

Our objective was to identify barriers to implementation of patient-centred approaches to AR management in APAC, and to propose solutions, paving the way for optimal management of AR by fully engaging the patient. A group of 11 expert otorhinolaryngologists and immunologists from Australia, Malaysia, the Philippines, Singapore, Thailand, and Vietnam provided expert insight into their experiences through a survey and discussions at a face-to-face meeting. These experts were selected on the basis of their experience in the management of patients with AR, as well as their interest in participating in this project. This paper presents the consolidated view of the practices of these experts in AR management and their suggestions on how to optimise patient-centred care across the patient journey for AR.

## Methods

The survey was conducted online and comprised 28 questions about the practices and experiences of the respondents in AR management. In addition to the survey, a worksheet containing 11 statements and recommendations regarding AR management, with a focus on patient-centred approaches was drafted. These sheets were provided prior to the face-to-face meeting, and experts were asked to report their agreement with each statement on a scale ranging from strongly agree to strongly disagree. The expert statements were derived based on existing guidelines and other information from the literature, as well as from previous interactions with experts.

The expert meeting covered challenges that may be faced in the implementation of patient-centred care at different stages of the patient journey and solutions to overcome them. At each stage, the survey results for that section were discussed, followed by a chaired discussion. Experts also drafted a visual representation of the patient journey and an antihistamine choice heatmap.

## Results

**Fig. 1:**
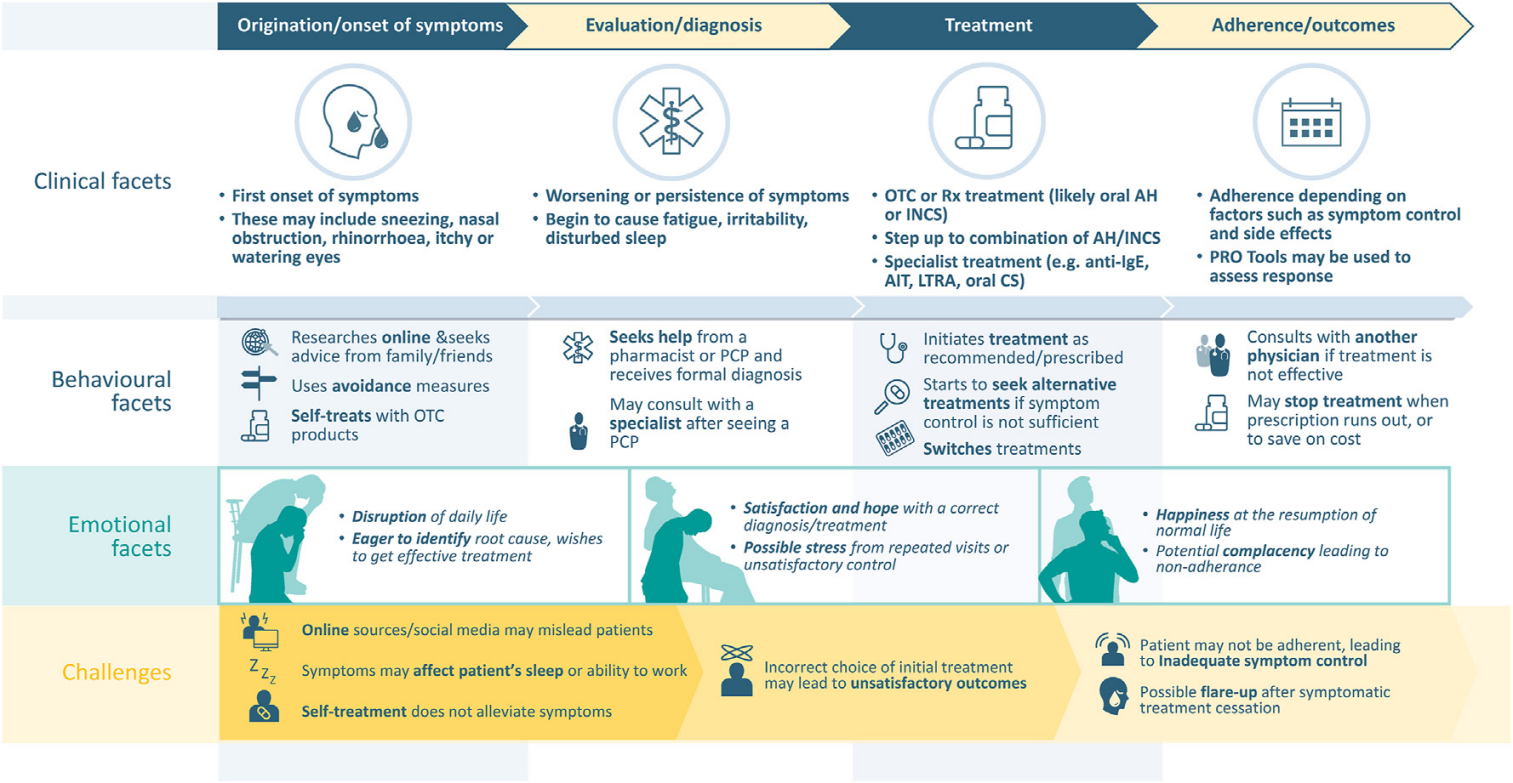
**Patient Journey for patients with allergic rhinitis or rhinoconjunctivitis**. Abbreviations: AH, antihistamine; AIT, allergen immunotherapy; CS, corticosteroid; ENT, ear nose and throat specialist; IgE, immunoglobulin E; INCS, intranasal corticosteroid; LTRA, leukotriene receptor antagonist; OTC, over-the counter; PCP, primary care provider; PRO, patient-reported outcome; QoL, quality of life; Rx, prescription VAS, visual analogue scale

The implementation of patient-centred approaches to AR management is important across the patient journey. This journey can be split into 4 stages: origination, evaluation/diagnosis, treatment, and fulfilment/adherence. It includes the clinical pathways for diagnosis and treatment found in guidelines, as well as the emotional and behavioural experiences of the patient. Importantly in the context of AR, it considers the experiences of the patient prior to their initial evaluation and diagnosis. A visual guide to this journey was developed to give context to the barriers described in this section (Fig. 1). The complete results of the survey used to gather information regarding expert practices are shown in Supplementary Figs. 1–5. Statements and recommendations agreed to by experts at the face-to-face meeting are presented in Table 2. The challenges that may be encountered in the implementation of patient-centred care in AR management, as well as proposed solutions are described in the following sections, with key barriers and solutions summarised in Table 3 and Table 4, respectively.

**Table 2:** Expert statements regarding the management of allergic rhinitis^a^

Statements	Expert responses to statements (n = 10^b^)				
	Strongly agree	Agree	Neutral	Disagree	Strongly disagree
AR negatively impacts the QoL of patients, affecting areas of the patient's life such as their sleep quality, education, and professional life.	10	0	0	0	0
For patient-centred care to be effective, patients should be educated about their condition and potential treatment options.	8	2	0	0	0
Further development of information sources such as printed materials would support the use of patient-centred care and shared decision-making in AR management.	7	3	0	0	0
AR management should be individualised for each patient in areas such as allergen avoidance and treatment choice.	10	0	0	0	0
To implement a patient-centred approach to care, the patient's specific health needs and desires should be understood and accounted for.	8	2	0	0	0
Second-generation antihistamine and/or intranasal corticosteroids (depending on severity) should be used as the first-line treatment for AR.	7	3	0	0	0
The use of first-generation antihistamines should be avoided due to their undesirable side effects.	5	3	1	1	0
Shared decision-making can support adherence and, where feasible, should be implemented when choosing treatments.	9	1	0	0	0
Understanding the patient's desires with regards to treatment outcomes is an important step in the implementation of patient-centred care.	9	1	0	0	0
PRO tools can provide useful information about treatment response and aid decision-making.	7	3	0	0	0
Symptom monitoring with PRO tools could help achieve optimal AR control.	6	3	1	0	0

Abbreviations: APAC, Asia-Pacific; AR, allergic rhinitis; CV, cardiovascular; PRO, patient-reported outcome; QoL, quality of life.

a The statements were conceived by the lead authors, with Medical Writing support.

b Note: one expert failed to return a copy of the statements and recommendations worksheet and therefore was not included in the counts presented in this table

**Table 3:** Key barriers to the implementation of patient-centred approaches to allergic rhinitis management in Asia-Pacific

Key barriers to the implementation of patient-centred care in Asia-Pacific
A lack of understanding or awareness of allergic rhinitis and its potential treatment options among patients.
Non-adherence to treatments recommended in guidelines.
Time or financial constraints that hinder the implementation of aspects of clinical guidelines such as the use of outcome measures in clinical practice.

**Table 4:** Key solutions to support the implementation of patient-centred approaches to allergic rhinitis management in Asia-Pacific

Key solutions that will support the implementation of patient-centred care in Asia-Pacific
Improved delivery of patient education explaining allergic rhinitis and its treatment options will support patient-centred care.
The implementation of shared decision-making to aid the choice of treatments that are most suitable for the patient and provide opportunities to support the patient's understanding of aspects of their treatments such as the need to take them continuously.
Patient characteristics and profiles should be considered when making treatment decisions to ensure that the most appropriate treatment is selected.
Patient-reported outcome measures should be used to gather information about treatment responses and aid decision-making

### Challenges to patient-centred management of allergic rhinitis

#### Origination and diagnosis

At the early stages of the patient journey (Fig. 1), a lack of awareness and understanding of AR and potential treatment options can be a barrier to patient-centred care. This barrier can be particularly relevant prior to diagnosis and referrals, with half (50%) of experts surveyed indicating that their patients are typically unaware of various aspects of AR at their first appointment, and a further 40% stating their patients would typically be aware of the disease but not of potential treatment options. Experts also reported that patients will often consult their families, research their condition online, and use over-the-counter treatments before consulting a healthcare provider. Additionally, the use of traditional or complementary medicines is not uncommon in APAC, with experts estimating that, on average, 10% of their patients would have tried them prior to referral.

With regard to educational materials, sources most commonly identified by experts as lacking in their country included patient advocacy groups (60% of experts), and printed materials (50% of experts). The availability of materials in appropriate languages for patients can also be an issue in APAC, with 40% of experts indicating that they are lacking, both in high- and middle-income countries (Australia, Malaysia, the Philippines).

#### Treatment and adherence

Once the patient has been diagnosed and started on treatment (Fig. 1), adherence can be a key issue. Experts agreed that measurement of adherence in AR management is typically difficult and often relies on patient interviews; there is a lack of devices such as dose-counters. Experts estimated on average that only 58% of their patients would be adherent to treatments. Low adherence can contribute to poor response to treatment, with the majority (80%) of experts surveyed identifying lack of adherence as one of the key causes of inadequate response. The reasons for non-adherence may vary. One common factor identified by experts was the type of treatment (60% of experts), with 60% naming intranasal corticosteroids as the most problematic treatment with regard to adherence, followed by first-generation antihistamines which were considered by 40% as the most problematic. Other factors that were identified as important in adherence were patient education (80% of experts), and severity of disease (80% of experts). Financial constraints may also affect AR management in APAC, with experts from Thailand and Vietnam reporting that economic factors may affect the choice of antihistamines in their countries. This is perhaps reflected in the continued use of first-generation antihistamines – 90% of experts reported some use of them amongst their patients (an average of 36% of patients) prior to referral.

#### Patient-reported outcomes

When asked about the use of patient-reported outcome (PRO) measures, time constraints were raised as a key barrier to their use in APAC, with 60% of experts reporting that insufficient time is the most common reason they decide not to use a QoL tool or other PRO measure. Non-adherence was also reported as an issue, with experts estimating that, on average, only 62% of their patients would complete PRO tools when offered them. Additionally, it was suggested that poor literacy may limit the use of PROs for some patients.

### Solutions that support patient-centred management of allergic rhinitis

#### Patient education

Education is crucial throughout the patient journey (Fig. 1), with all experts agreeing that patients should be educated about their condition and treatment options in order for patient-centred care to be effective (80% strongly agree, 20% agree; Table 2). It was also agreed that the further development of information sources for patients in APAC would support the implementation of patient-centred approaches to AR management (70% strongly agree, 30% agree; Table 2). Key points raised in the face-to-face meeting that should be addressed in such educational materials are identifying the symptoms of AR, available management options, and when to consult a physician. Other important factors to convey to patients included the importance of continuing treatment and allergen avoidance, an understanding of whether they are on the correct path, and of management costs. It was agreed that this information should be provided in a simple format and should be standardised where possible, with materials for paediatric patients tailored for children and their carers. A variety of methods for the dissemination of this information were suggested, including leaflets in general practitioner clinics/pharmacies, hospital websites, or links/QR codes.

It was added that, as patients will often first seek care from pharmacists and general practitioners, it is important that these individuals are educated on how to make a correct diagnosis. Minimal diagnostic criteria identified by the experts included persistent symptoms (>2 weeks; including runny nose, sneezing, and itchy eyes), potentially seasonal worsening, and a response to antihistamines, in addition to a lack of fever, sore throat, or other common cold symptoms. Proposed criteria for referrals included atypical symptoms, a lack of first-line treatment response, the presence of other significant atopic disease, or if the patient expresses a preference for seeing a specialist.

#### Patient-centred approaches to treatment

**Fig. 2:**
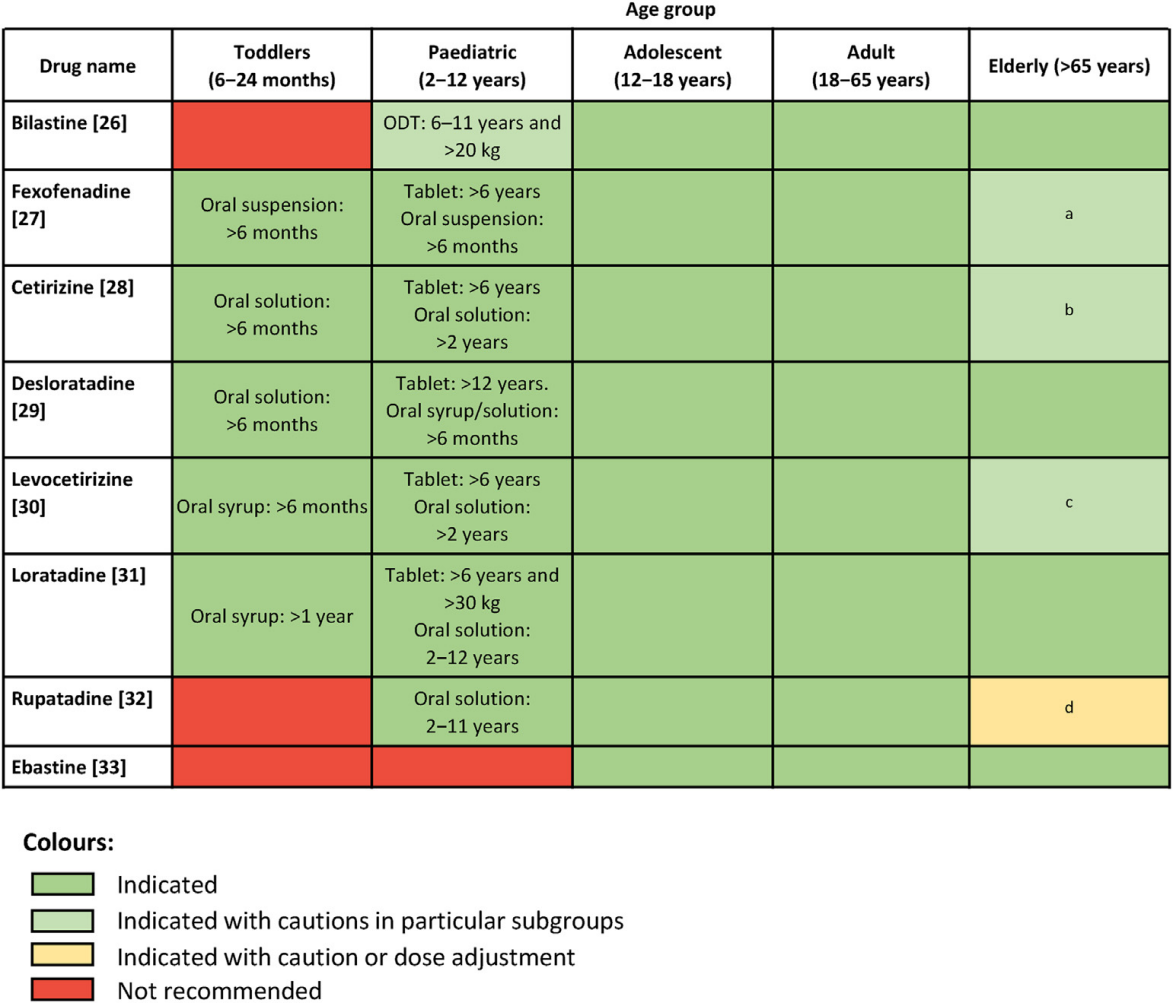
**Visual guide to the choices of antihistamines for different age groups**. Abbreviation: ODT, orodispersible tablet. This heatmap was drafted using the prescribing information for each included antihistamine^26^, ^27^, ^28^, ^29^, ^30^, ^31^, ^32^, ^33^ in addition to input from both the allergic rhinitis and urticaria STAR-Network expert groups. ^a^Care should be taken in dose selection due to the potential for renal impairment. ^b^Caution in elderly patients with renal/hepatic impairment.^28^^c^With adjustment for renal function.^30^^d^Use with caution in elderly patients^32^

**Fig. 3:**
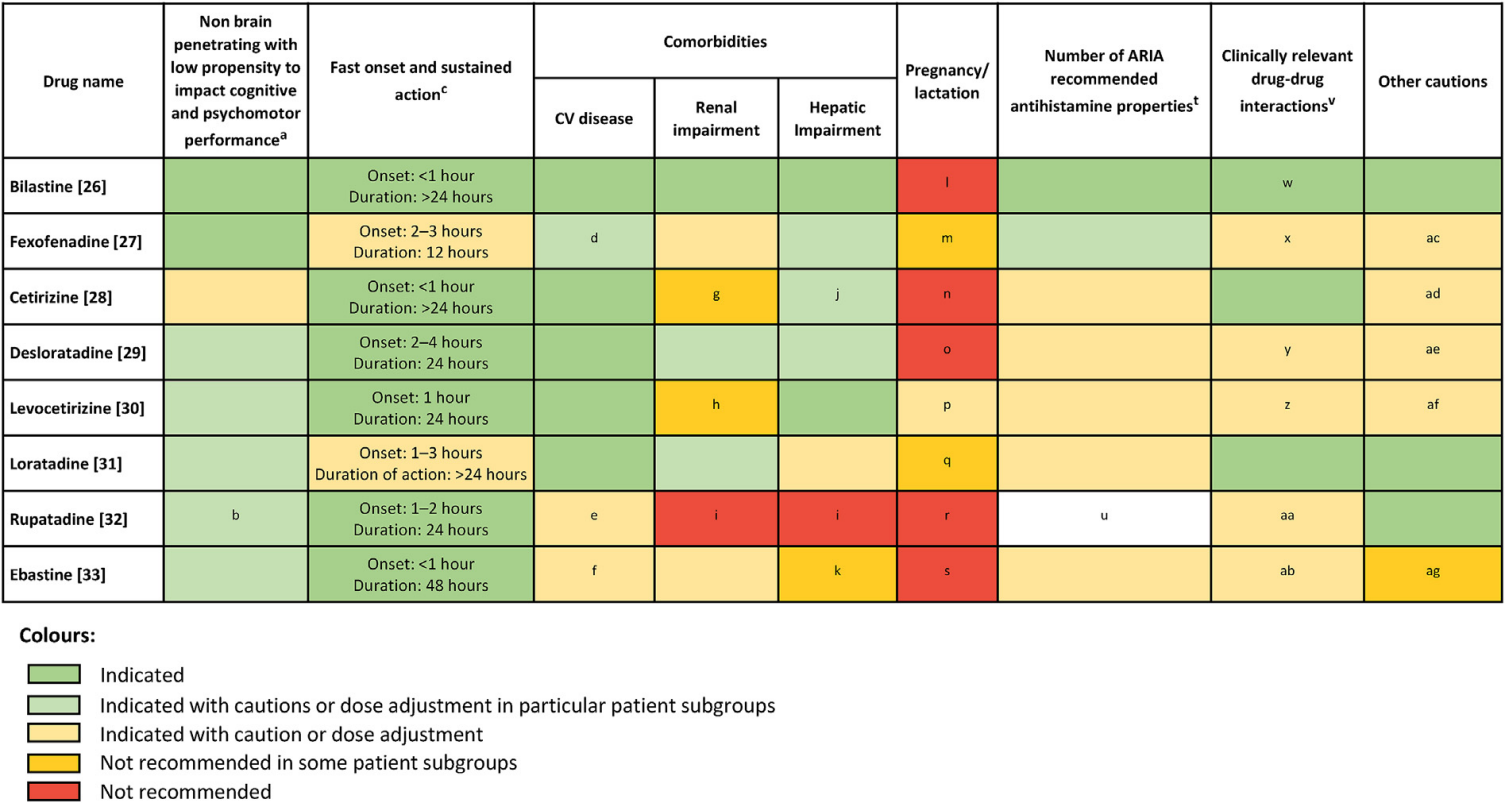
**Visual guide to antihistamine choices from patient-centred criteria**. Abbreviations: ARIA, Allergic Rhinitis and its Impact on Asthma; CV, cardiovascular. This heatmap was drafted using the prescribing information for each included antihistamine^26^, ^27^, ^28^, ^29^, ^30^, ^31^, ^32^, ^33^ in addition to input from both the allergic rhinitis and urticaria STAR-Network expert groups. ^a^Colours based on the classification of these antihistamines by H1 receptor occupancy in Kawauchi et al.,^34^ with green being non-brain penetrating, pale green being non-sedating, and yellow being less sedating. ^b^H1 receptor occupancy data are not available for rupatadine. However, it is classified as non-sedating based on patient-reported scales and driving tests.^35^^c^As reported for histamine skin wheal studies in prescribing information/package inserts. Coloured according to time values. ^d^Patients with CV disease or a history of CV disease receiving fexofenadine should be alerted to the fact that antihistamines as a therapeutic group have been associated with the undesirable effects of tachycardia and palpitations.^27^^e^Should be used with caution in patients with known prolongation of the QT interval, uncorrected hypokalaemia, or ongoing proarrhythmic conditions^32^. ^f^Should be used with caution in patients with prolonged QT interval, hypokalaemia, or with concomitant use of QT-prolonging agents^33^. ^g^Dose adjustments required and contraindicated with severe renal impairment^28^. ^h^Dose adjustments required. Contraindicated in severe renal impairment^30^. ^i^The use of rupatadine is not presently recommended in patients with impaired kidney or liver functions due to a lack of clinical experience in these patients. ^j^Dose adjustments are recommended for some forms of cetirizine in patients with hepatic impairment. ^k^Contraindicated with severe liver failure. Caution with severe hepatic impairment. ^l^As a precautionary measure, it is preferable to avoid the use of bilastine during pregnancy. ^m^Fexofenadine should only be used during pregnancy when the potential benefits justify the possible risks to the foetus. ^n^Preferable to avoid use during pregnancy, contraindicated/caution for lactation. ^o^Preferable to avoid in pregnancy, not recommended in lactation. ^p^The use of levocetirizine may be considered during pregnancy, if necessary. Caution should be exercised when prescribing levocetirizine to lactating women. ^q^Should only be used if benefits outweigh potential risks. ^r^As a precautionary measure it is preferable to avoid the use of rupatadine during pregnancy. ^s^Not recommended in pregnant or lactating women. ^t^As reported in Wang et al.^36^ Coloured according to score with 10 as green, 7–10 as pale green, and <7 as yellow. ^u^A score for rupatadine was not available in Wang et al.^36^. ^v^As reported in package inserts and prescribing information. ^w^Plasma concentration may be decreased by OATP1A2 substrates or inhibitors. Avoid coadministration of bilastine and P-glycoprotein inhibitors in patients with moderate or severe renal impairment. ^x^Caution with sedative use. ^y^Erythromycin/ketoconazole may increase plasma levels. Aluminium/magnesium-containing antacids decrease AUC and Cmax if administered at a similar time. ^z^Concomitant use with central nervous system suppressants should be avoided. Decreased clearance of cetirizine with theophylline. Increased exposure of levocetirizine with ritonavir. ^aa^Avoid use with strong CYP3A4 inhibitors. Caution when co-administering with statins. Concomitant administration with ketoconazole or erythromycin increases systemic exposure to rupatadine. ^ab^Caution with QT-prolonging agents, hepatic enzyme inhibitors (CYP450 2J2, 4F12, 3A4) such as imidazole antifungals or macrolide antibiotics. Care should be taken with imidazole antifungals, macrolide antibiotics, and antituberculosis drugs. ^ac^Caution in epileptic patients and patients with predisposition to urinary retention. ^ad^Caution should be used in patients with history of seizures and patients with predisposition to urinary retention. ^ae^Caution should be used in patients with a history of seizures. ^af^Caution in epileptic patients and patients with predisposition to urinary retention. ^ag^Not for use in patients with rare genetic disorders of galactose intolerance, Lapp lactase deficiency, or glucose-galactose absorption disorders

All experts agreed that the implementation of patient-centred care at the treatment stage of the patient journey (Fig. 1) involves accounting for the patient's specific health needs and desires (80% strongly agree, 20% agree; Table 2), particularly when choosing treatments. These may vary between patients, but experts indicated that typical desires include reduction in nasal symptoms (70% of experts), avoidance of side effects (60% of experts), and complete resolution of symptoms (50% of experts). These wishes, as well as other patient characteristics or profiles should therefore be considered when choosing medications, as has previously been recognised for antihistamines.^19^^,^^20^ To aid choosing the most appropriate second-generation antihistamine, heatmaps providing a visual representation of the suitability of different antihistamines for patients with particular characteristics were developed using information from local prescribing information and expert insight (Fig. 2, Fig. 3). Fig. 2 is a guide to the suitability of antihistamines for different age groups. Fig. 3 addresses a variety of other factors that may be relevant to the patient, including comorbidities, sedation, and onset and duration of action.

The use of SDM may encourage adherence by both ensuring that these preferences are met, and by providing opportunities to help patients understand the need to adhere to their treatment. All experts agreed that SDM can support adherence and, where feasible, should be implemented when choosing treatments (90% strongly agree, 10% agree; Table 2). It was also suggested that in cases where adherence is poor, the reasons should be explored with the patient and addressed where possible. Additionally, it was raised that it may be worth re-evaluating the diagnosis to ensure the patient is receiving the correct treatment, and that SDM should then be used when deciding on the next steps.

#### Patient-reported outcome tools

The use of PRO tools such as the visual analog scale (VAS) should be considered throughout the patient journey (Fig. 1), with all experts agreeing that such tools can aid AR management by providing feedback on the patient's response to treatment (70% strongly agreed, 30% agreed; Table 2). These tools may also support patient-centred care by helping patients to more specifically express their desires regarding outcomes, as stated by half (50%) of the experts surveyed. It was suggested that such tools should be used at baseline, 1−2 weeks after treatment initiation, and then whenever possible during maintenance treatment; mild/moderate/severe classifications or symptom diaries could be used as an alternative to numerical scales such as VAS, if preferred. Mobile applications were agreed to be of potential benefit in AR management, with 90% of experts agreeing that an app to monitor AR symptoms and their effects on QoL would be useful in their country. However, it was raised that, although existing apps such as MASK-air may fulfil these roles,^10^ administrative and language issues may complicate their use in APAC. Regardless of the form of outcome measure used, experts agreed that it is important to allow sufficient time to elapse before assessing if the response to a treatment is inadequate and suggested that two weeks should be considered the minimum time before assessment. It was also pointed out that, if outcome measures are to be used to support adherence, they should be implemented in a manner that allows the patient to see their data or receive feedback on their results.

## Discussion

The substantial burden of AR to patients^3^^,^^6^^,^^7^ and to society^3^^,^^8^ persists, despite the availability of efficacious treatments and numerous recommendations for diagnosis and treatment in international guidelines. It is increasingly recognised that there are gaps in the implementation of those recommendations into patient care. Patient-centred approaches to care may aid in reducing these gaps and have previously been shown to be beneficial in other disease areas in improving adherence,^15^ outcomes, and patient satisfaction.^13^ Such approaches are also already incorporated into some national guidelines for AR management. For example, guidelines published by the American Academy of Otolaryngology–Head and Neck Surgery include specific statements regarding the role of patient preferences and the use of SDM for particular treatments and diagnostic techniques.^21^

We have identified specific barriers that may be encountered across the patient journey in the context of AR management (Table 3). At the early stages of the patient journey, a lack of awareness of AR and potential treatment options can impede the patient's access to care. The use of traditional or complementary therapies is more or less common in APAC, with studies finding that 62.1% of participants with AR in Malaysia had used complementary/alternative medicines in the last 10 years^22^ and 18.1% and 0.5% of patients had used traditional medicine in 2011 in Taiwan and South Korea, respectively.^23^ The participating experts indicated that more than one third of their patients used first-generation antihistamines prior to referral. At later stages, poor adherence can be a particular issue in AR management, with one global study reporting that only 11% of patients with AR are adherent while 4% are partly adherent.^24^ Other maintenance barriers may include financial constraints on treatment options, and time constraints that hinder aspects of care such as the use of outcome measures.

We propose several solutions to support the implementation of patient-centred care, based on expert opinion and current evidence (Table 4). Patient education is crucial throughout the patient journey to facilitate the implementation of patient-centred care and support adherence. As patients with AR may live with their condition for extended periods prior to diagnosis and self-manage their symptoms, the provision of information online or through pharmacies may be of particular importance. When choosing treatments, patient needs and desires should be accounted for, and SDM should be used to facilitate the choice of the most appropriate treatment. The importance of considering patient characteristics and profiles when prescribing antihistamines has been highlighted previously,^19^^,^^20^ and we have produced a visual guide to support decision-making. Outcome measures should also be used when feasible, as recommended in international guidelines,^10^ to gather feedback on treatment responses and further facilitate the optimisation of care. Areas for further development and optimisation include the forms in which educational materials and outcome measurement tools are provided to patients.

In this manuscript, we have presented the views of experts representing 6 APAC countries, with a combined population of 351 million.^25^ Experts from India and China were invited, but they did not participate, and further work will be required to identify challenges and solutions specific to these countries. It is also worth noting that, due to the limited number of experts surveyed, not all issues discussed here will necessarily be generalisable to all practices or apply equally to all parts of the region. For example, patients in Australia more commonly report visiting pharmacists and using over-the-counter medications in addition to fewer specialist visits than patients in other countries in Asia.^3^ A country's income level (2 high-income, 2 upper middle-income and 2 lower-middle income countries are included), as well as its particular healthcare system will play an important role in how our findings can be successfully applied, which should be explored in further research. Furthermore, as this study has focussed on patient-centred approaches to care, we have not explored all treatment options for AR in detail. Treatments such as intranasal antihistamines and corticosteroids are important components of AR management and should be considered when implementing patient-centred approaches to care. In APAC, there is currently little focus on patient-centred approaches to care, which have the potential to greatly improve the management of AR. The work presented here suggests action points for general practitioners, specialists, and healthcare systems.

## Abbreviations

APAC, Asia-Pacific; AR, allergic rhinitis; IgE, immunoglobulin E; PRO, patient-reported outcome; QoL, quality of life; SDM, shared decision-making; VAS, visual analogue scale.

## Funding

This study was sponsored by A. Menarini Asia Pacific Pte Ltd. Medical writing support was provided by Clarivate and funded by A. Menarini Asia Pacific Pte Ltd.

## Data and materials availability

Data from the questionnaire are included in full in the supplementary materials. All other data or materials are available from the corresponding author upon reasonable request.

## Author contributions

All authors contributed to the development of the manuscript at a face-to-face meeting and reviewed the final draft of the manuscript. HC, DYW, and DN contributed to and reviewed the questionnaire, meeting materials, and initial drafts of the manuscript.

## Authors' consent for publication

All authors have agreed with the publication of this manuscript in the World Allergy Organization Journal.

## Submission declaration

This manuscript is original, has not been published before, is not currently being considered for publication elsewhere.

## Declaration of competing interest

All authors received honoraria and support from A. Menarini Asia Pacific Pte Ltd for transportation and accommodation for the STAR Network meeting. A. Menarini also provided support staff at the meeting and funded medical writing support from Clarivate for the development of the manuscript. HC also reports the following outside of the submitted work: payment or honoraria for lectures from GSK, Takeda, A. Menarini, Sanofi, and Novartis. DP also reports the following outside of the submitted work: payment or honoraria for lectures; support for attending regional advisory board meetings; Medical directorship of the AMSI Doctors Medical Center in the Philippines; founder and stockholder of the AMSI Doctors Medical Center, Philippines. PT also reports the following outside of the submitted work: payment or honoraria for lectures for GSK.DN works with A. Menarini Asia Pacific Pte Ltd and was involved in facilitating the expert meeting. The remaining authors report no additional disclosures outside of the submitted work.
