# Supplementary material for: Promoting patient-centred care in the management of allergic rhinitis in Asia-Pacific countries
Source: World Allergy Organ J. 2024 Aug 22;17(9):100952. doi: 10.1016/j.waojou.2024.100952 (PMC11388691; doi:10.1016/j.waojou.2024.100952)
Supplement: Multimedia component 1 [file mmc1.pdf]

Promoting patient-centred care in the management of allergic rhinitis in Asia-Pacific countries: supplementary materials

Supplementary figure 1: Survey results for questions on origination and patient awareness (n=10 respondents).

Q1 - Approximately how many patients do you see in a typical month with mild AR, and with moderate-severe AR?

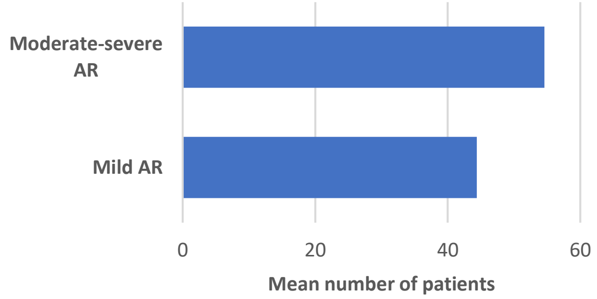

Q2 - Approximately how many patients do you see in a typical month with intermittent AR, and how many do you see with persistent AR?

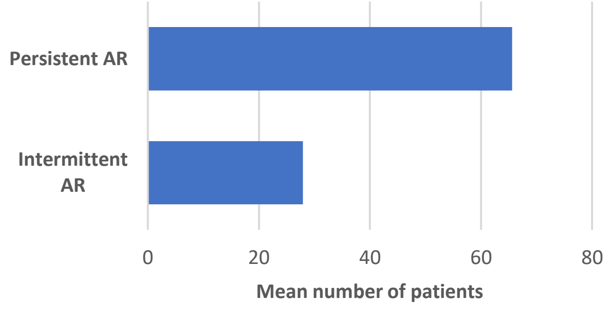

Q3 - How aware are your patients of AR and possible treatment options before their first appointment with you?

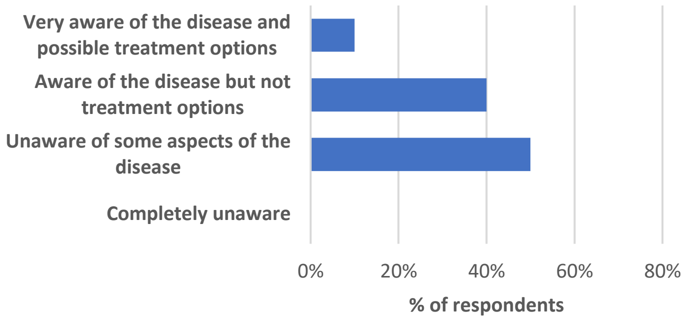

Q4 - What resources about AR would they typically have accessed before seeing you?

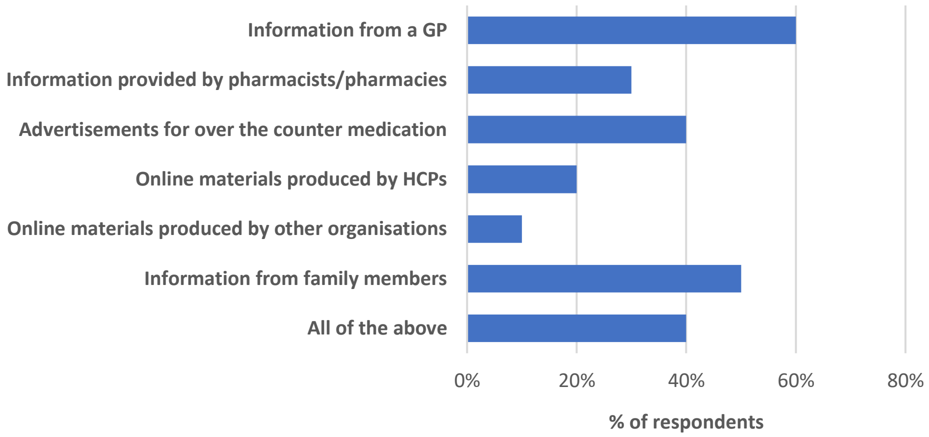

Q5 - Which educational materials for patients visiting the clinic do you have, to support them in understanding their diagnosis and treatment options?

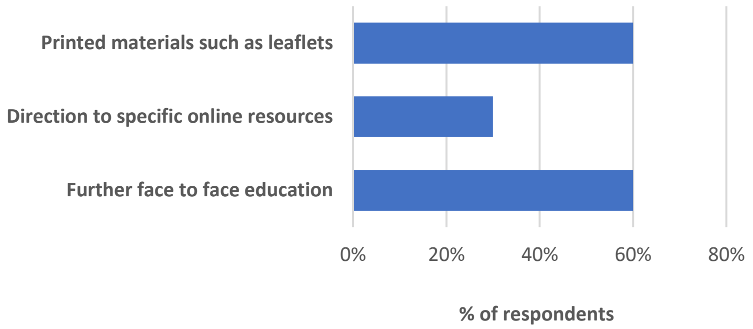

Q6 - Which of the following types of educational material are lacking in your country or could be substantially improved?

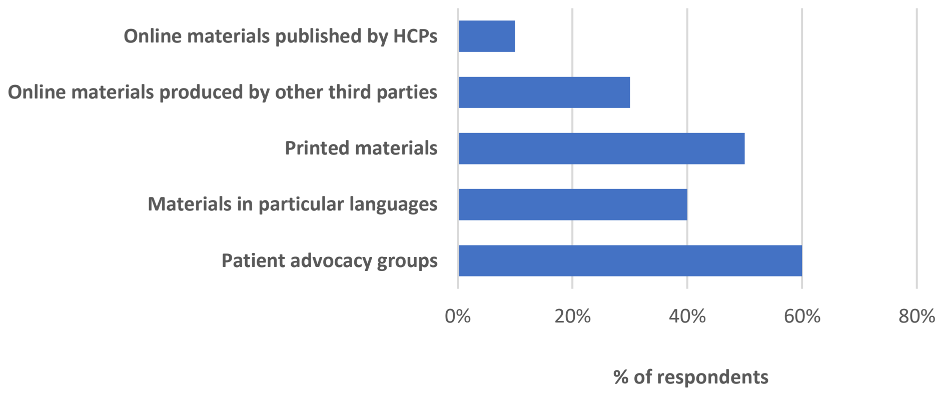

Abbreviations: AR, allergic rhinitis; GP, general practitioner; HCP, healthcare providers.

Supplementary figure 2: Survey results for questions on evaluation and diagnosis (n=10 respondents).

Q7 - How do patients normally get an appointment with you?

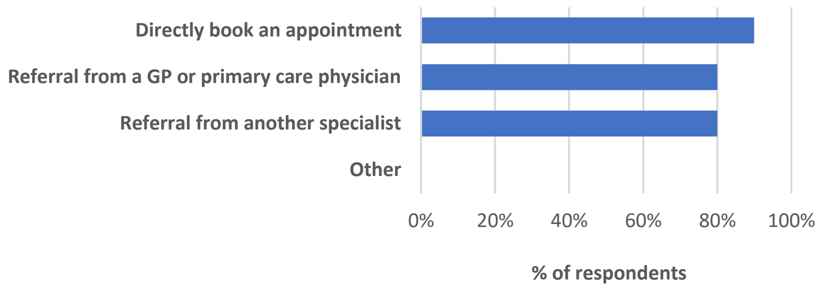

Q8 - In your practice, what would be common reasons for referral of a patient with AR? Please provide percentages.

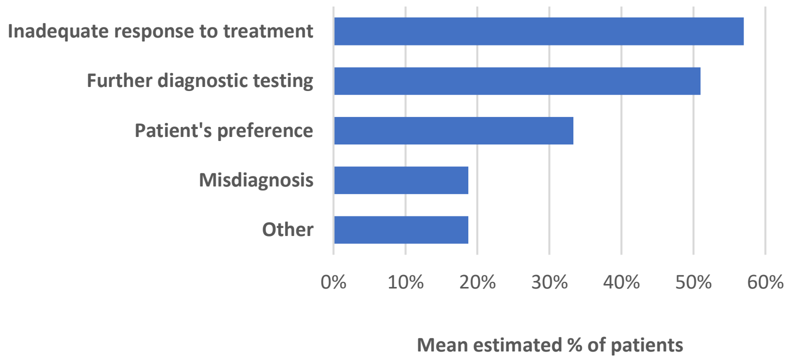

Q9 - Do you feel any of your patients could have been better managed in primary care prior to referral (e.g. due to inadequate dosing in primary care)?

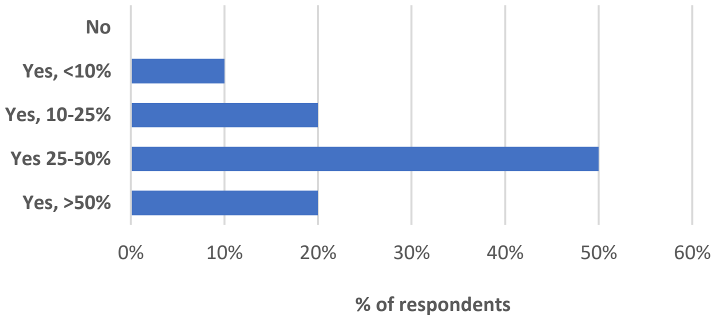

Q10 - Approximately how many of your patients would have self-treated before seeing you?

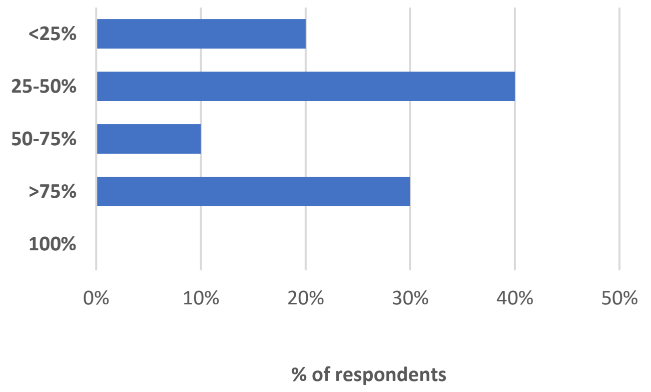

Q11 - Approximately how many of your patients would have received a prescription from a GP before seeing you?

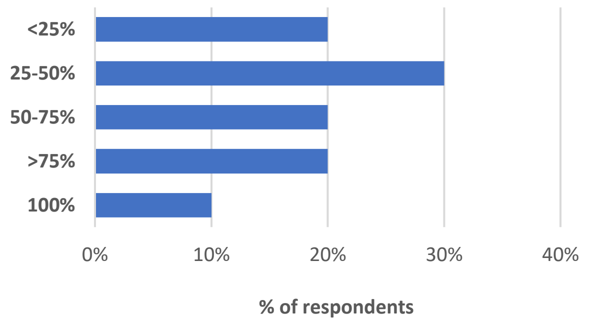

Q12 - What treatment options would patients commonly have tried before being referred?

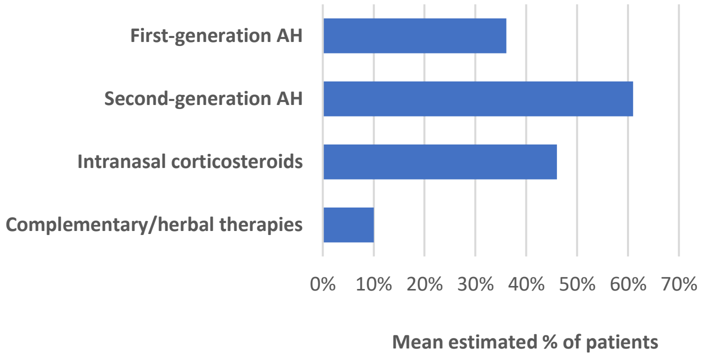

Abbreviations: AH, antihistamine; GP, general practitioner.

Supplementary figure 3: Survey results for questions on treatment (n=10 respondents).

Q13 - Which treatment do you usually recommend/prescribe at the patient's first visit?

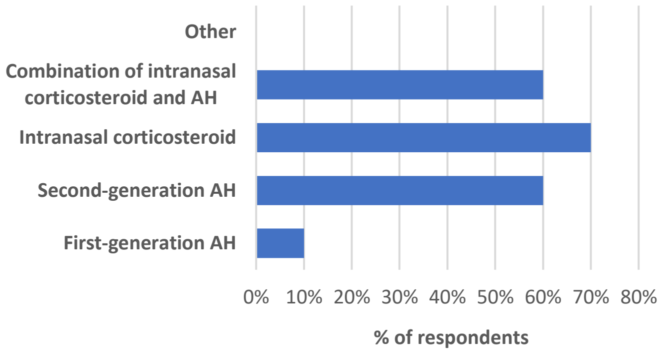

Q14 - How many of the patients you treat first-line have an inadequate response?

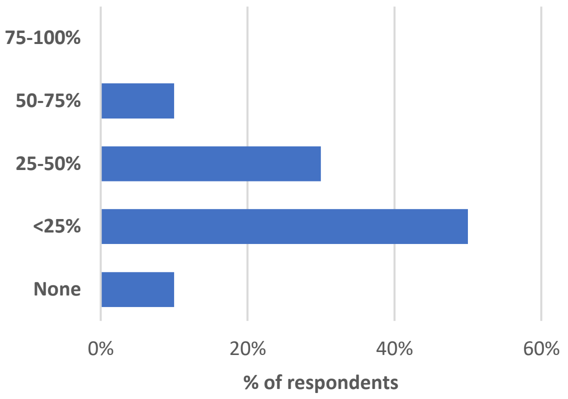

Q15 - What are the main reasons for/drivers of inadequate response?

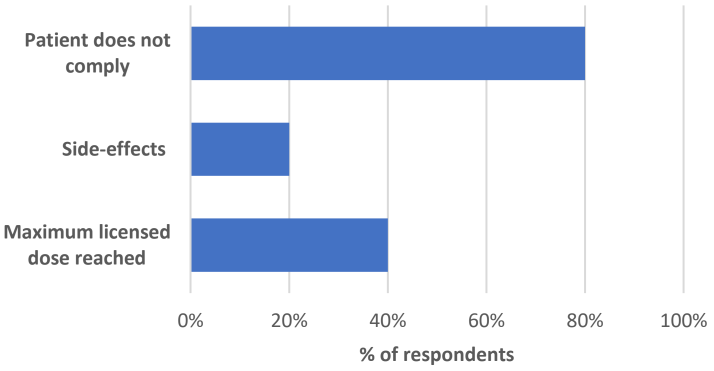

Q16 - When there is inadequate response, do you:

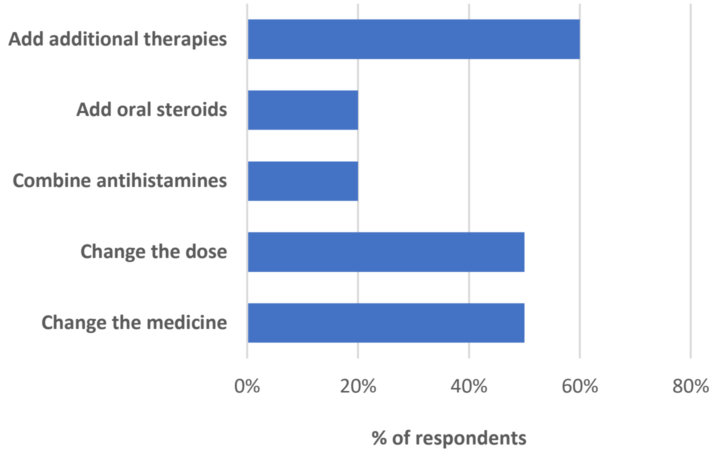

Q17 - Which treatment do you usually prescribe second-line, following inadequate response to the first-line treatment?

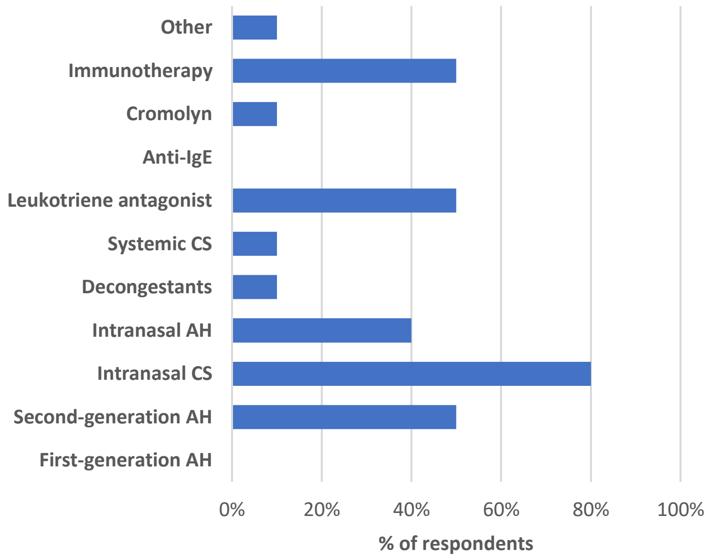

Q18 - If you prescribe second-generation antihistamines, what factors affect your choice of antihistamine?

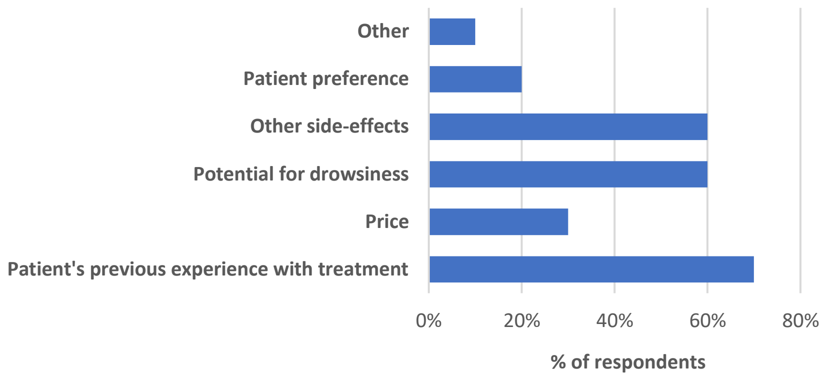

Q19 - Do you regularly prescribe bilastine, and if so, which of the following patients do you prescribe it to?

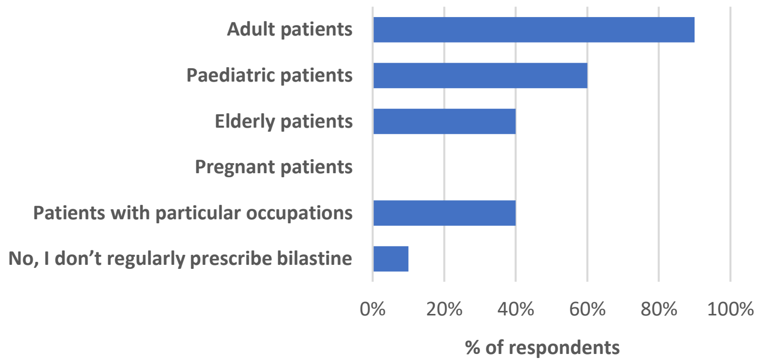

Q20 - Which of the following outcomes do you think are the most important to patients?

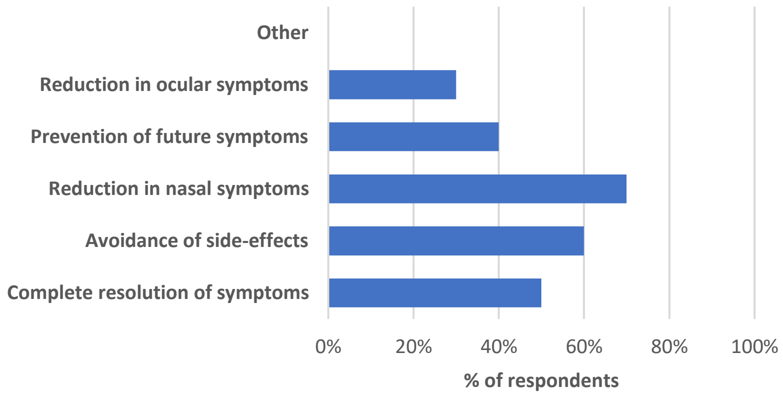

Abbreviations: AH, antihistamine; CS, corticosteroids; IgE, immunoglobulin E.

Supplementary figure 4: Survey results for questions on compliance (n=10 respondents).

Q21 - In your experience, approximately what percentage of patients are adherent to their treatments?

Respondents estimated a mean of 58.33% of patients are adherent to their AR treatments.

Q22 - Which of the following treatments, if any, are typically the most problematic with regard to compliance?

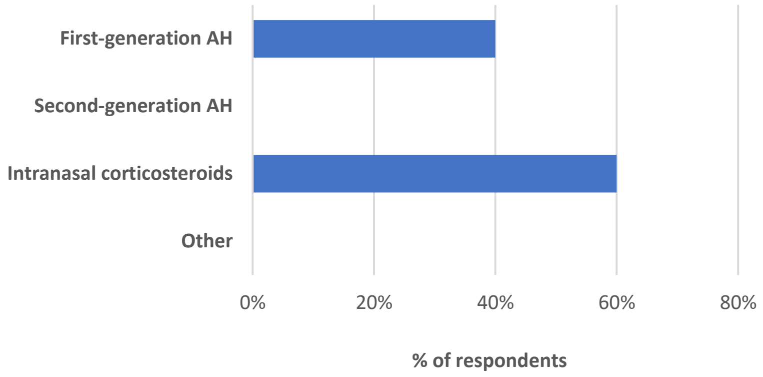

Q23 - Which of the following are typically the most important factors in determining whether a patient is compliant?

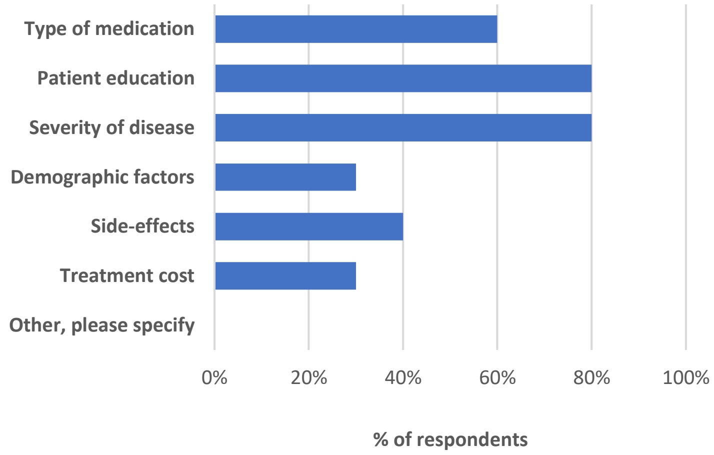

Abbreviation: AH, antihistamine.

Supplementary figure 5: Survey results for questions on outcomes (n=10 respondents).

Q24 - Do you regularly use patient reported outcome tools (e.g. TNSS, VAS) with patients with AR?

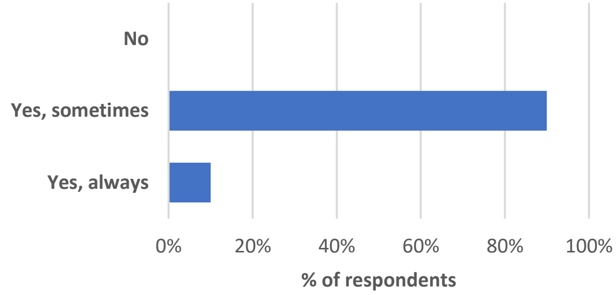

Q25 - If you have offered to use outcome measurements and tools with patients, approximately what percentage of patients with AR complete them?

Respondents estimated a mean of 62.2% of their patients complete outcome measures when offered them.

Q26 - Which of the following would be the most common reason you decide not to use a quality of life tool or other outcome measure with a patient?

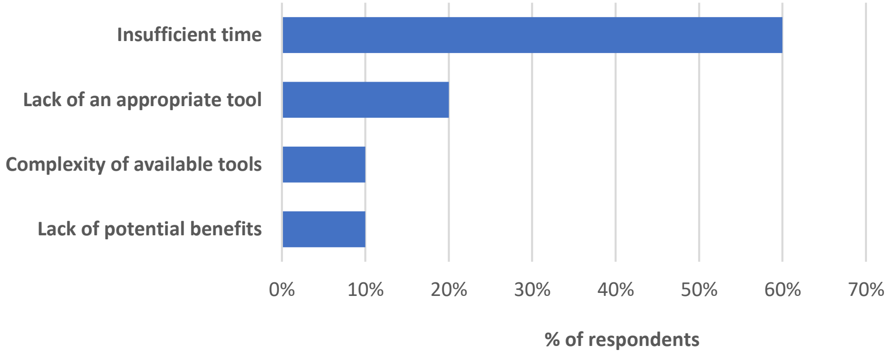

Q27 - When you have used outcome measures, which of the following aspects have you found useful?

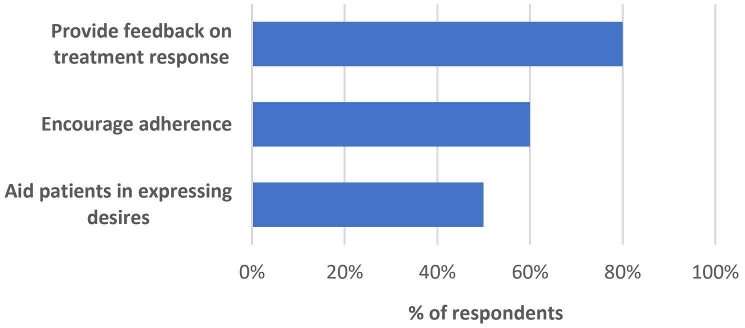

Q28 - Would the development of an app to monitor AR symptoms and their effects on quality of life be a useful step-forward in AR management in your country?

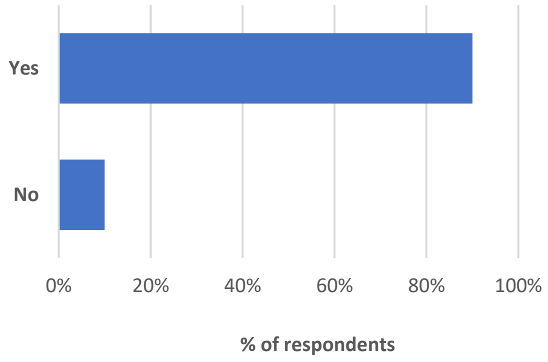

Abbreviations: AR, allergic rhinitis; TNSS, Total nasal symptoms score; VAS, visual analogue scale.
